# Neurocognitive Adverse Events Related to Lorlatinib in Non-Small Cell Lung Cancer: A Systematic Review and Meta-Analysis

**DOI:** 10.3390/cancers16142611

**Published:** 2024-07-22

**Authors:** Jonathan N. Priantti, Maysa Vilbert, Francisco Cezar Aquino de Moraes, Thiago Madeira, Evair Moisés de Lima Santiago, Natasha B. Leighl, Ludimila Cavalcante, Nagla F. Abdel Karim

**Affiliations:** 1 School of Medicine, Federal University of Amazonas—UFAM, Manaus 69020-160, AM, Brazil; jonathan.priantti@gmail.com; 2 Massachusetts General Hospital Cancer Center, Division of Hematology/Oncology, Department of Medicine, Massachusetts General Hospital, Boston, MA 02114, USA; 3 School of Medicine, Federal University of Pará—UFPA, Belém 66050-060, PA, Brazil; 4 School of Medicine, Federal University of Minas Gerais—UFMG, Belo Horizonte 30130-100, MG, Brazil; 5 School of Medicine, Federal University of Mato Grosso do Sul—UFMS, Campo Grande 79070-900, MS, Brazil; 6 Princess Margaret Cancer Centre, University Health Network, Toronto, ON M5G 2C4, Canada; 7 Department of Medical Oncology and Hematology, University of Virginia Comprehensive Cancer Center, Charlottesville, VA 22903, USA; 8 Inova Schar Cancer Institute, University of Virginia, Fairfax, VA 22031, USA

**Keywords:** adverse effects, lorlatinib, lung cancer, ALK, ROS1

## Abstract

**Simple Summary:**

This systematic review and meta-analysis aimed to evaluate the neurocognitive adverse events (NAEs) related to lorlatinib in patients with ALK/ROS1-positive non-small cell lung cancer (NSCLC). A random-effects model was used for the meta-analysis and the proportions were pooled with 95% confidence intervals (CIs). In our pooled analysis, cognitive and mood effects were the most important NAEs, with 14% and 11% overall rates, respectively. Speech changes and psychotic effects had an overall frequency of 7% and 5%, respectively. In a subanalysis, mood effects were significantly more frequent in clinical trials than in real-world studies. Therefore, this study showed that lorlatinib-related NAEs are emerging important toxicities in clinical practice. Hence, increasing awareness about NAEs is paramount to improving their recognition and reporting, and thus enhancing patients’ quality of life.

**Abstract:**

Lorlatinib has been FDA-approved as a systemic therapy for ALK/ROS1-positive non-small cell lung cancer (NSCLC) patients. However, it has been associated with an increased frequency of neurocognitive adverse events (NAEs). Therefore, we conducted a systematic review and meta-analysis to assess the NAEs related to lorlatinib therapy in NSCLC patients. PubMed, Scopus, the Cochrane Library, and prominent conference proceedings were searched for eligible studies of lorlatinib in NSCLC patients. NAEs included cognitive, mood, speech, and psychotic effects. A total of 1147 patients from 12 studies were included; 62% had brain metastases. A pooled analysis of NAEs showed frequencies of cognitive effects of 14.57% (95% CI, 8.37 to 24.14, I2 = 84%), mood effects of 11.17% (95% CI, 5.93 to 20.07, I2 = 84%), speech effects of 7.24% (95% CI, 3.39 to 15.20, I2 = 72%), and psychotic effects of 4.97% (95% CI, 3.27 to 7.49, I2 = 21%). Clinical trials reported a significantly higher frequency of mood effects than was indicated by real-world data. These results highlight the importance of educating patients and healthcare professionals about lorlatinib-related NAEs for early detection and management to improve NSCLC patients’ quality of life.

## 1. Introduction

Lorlatinib, a third-generation tyrosine kinase inhibitor (TKI), offers several advantages over early-generation TKIs, with a high selectivity for ALK/ROS1 genetic aberrations [1,2,3,4,5]. Beyond providing an excellent central nervous system (CNS) response, lorlatinib has activity against common resistance mechanisms and was first used after failure of prior TKIs [5]. Most recently, the CROWN trial has established lorlatinib as a first-line treatment option, owing to its superior efficacy in preventing and treating intracranial metastasis and eliciting great tumor responses [4,5,6,7,8].

Notwithstanding its superior anti-tumor efficacy and survival benefit, lorlatinib causes a distinct range of adverse events when compared to other TKIs, such as CNS toxicity, represented by a variety of new neurological symptoms [7,9,10]. These adverse effects, collectively referred to as neurocognitive adverse effects (NAEs), are linked to impairment in mood, cognition, and speech, and include psychosis [7,9,10].

In the CROWN study, approximately 40% of patients developed NAEs, albeit most of them were grade 1 or 2 [8]. Interestingly, real-world studies demonstrated different incidences of this specific toxicity profile, varying from 3% to 32% among cohorts [11]. In addition, previous high-quality systematic reviews and meta-analyses have not evaluated the wide spectrum of these lorlatinib-related NAEs [12,13]. Furthermore, as there is little in the way of objective standards for identifying and quantifying these effects in cancer patients, there is a need for heightened awareness of NAEs by multidisciplinary healthcare teams, patients, and families [14,15]. Hence, this systematic review and meta-analysis aimed to investigate lorlatinib-related NAEs, establish their frequency amongst studies (clinical trials and real-world evidence), and search for possible associated risk factors.

## 2. Materials and Methods

The study protocol was registered in PROSPERO (CRD42023417365) and was conducted according to the Cochrane Collaboration and the Preferred Reporting Items for Systematic Reviews and Meta-Analysis (PRISMA) statement guidelines (PRISMA Checklist, Supplementary Tables S1 and S2) [16].

### 2.1. Eligibility Criteria

This systematic review and meta-analysis included (1) randomized and nonrandomized clinical trials, as well as prospective or retrospective observational cohort studies; (2) patients diagnosed with ALK-positive or ROS1-positive advanced or metastatic NSCLC who were treated with lorlatinib (3) reporting any NAEs within the categories of “cognitive effects”, “mood effects”, “speech effects”, and “psychotic effects”.

Studies (1) with no outcomes of interest; (2) that were case reports, case series, or case-control studies; (3) combining lorlatinib with another drug in the investigational arm; or (4) with overlapping populations were excluded.

### 2.2. Search Strategy

PubMed, Scopus, and the Cochrane Central Register of Controlled Trials were systematically searched for eligible studies. Additionally, the official websites of the American Society of Clinical Oncology, the European Society for Medical Oncology, and the American Association for Cancer Research were included in the search.

The search query encompassed the terms “lorlatinib” and “non-small cell lung cancer”. Additionally, the Boolean operator “AND” was employed to combine the search terms in each database, except for congress/conference websites, where only the term “lorlatinib” was used. The search contained the available literature up until May 2023. Furthermore, a manual search was performed to identify any additional studies referenced in previous systematic reviews and/or meta-analyses.

### 2.3. Data Extraction

Data extraction was carried out by two authors (J.N.P and F.C.A.M) according to predetermined search criteria and quality assessment protocols. In cases where a consensus regarding study inclusion was not reached, the determination of its eligibility was made by a third author, M.V.

### 2.4. Endpoints and Subanalysis

Neurocognitive adverse events (NAEs) included in this meta-analysis were defined as any adverse effect characterized based on the Common Terminology Criteria for Adverse Events (CTCAE) and comprising the cluster term definitions extracted from all Preferred Terms under the Medical Dictionary for Regulatory Activities (MedDRA) Queries, shown in Supplementary Table S3.

Prespecified subgroup analysis included the frequency of neurocognitive adverse events considering ethnicity (Asians versus non-Asian patients), mutation status (ALK-positive versus ROS1-positive), CNS disease (presence versus absence of brain metastasis), and type of study (clinical trials versus observational studies).

In the subgroup analysis characterized by the presence or absence of CNS disease, other symptoms associated with the CNS, such as dizziness, daze, headache, dysgeusia, insomnia, and vision disorders were also included.

### 2.5. Quality Assessment

Nonrandomized interventional studies were evaluated by the Risk of Bias in Non-randomised Studies-of Interventions (ROBINS-I) tool [17]. The risk-of-bias assessment tool for randomized trials (RoB 2) developed by the Cochrane Collaboration [18] was used for the evaluation of the quality of randomized studies. We used the Newcastle–Ottawa Scale (NOS) [19] to assess the methodological quality of the observational studies.

### 2.6. Statistical Analysis

We used RStudio (Posit Software, PBC, version 2022.12.0+353) and R (version 4.2.2) for statistical analysis, and we pooled the rates with 95% CI proportions. We used logit transformation of data when an individual study proportion was <20% or >80%. Additionally, in the case of a study with zero events or with an individual proportion of 100%, the Freeman–Tukey double arcsine transformation was used [20,21]. We applied a DerSimonian and Laird random-effects model to all analyses. We set the statistical significance threshold at a *p*-value ≤ 0.05. Heterogeneity was assessed with I^2^ statistics. Funnel plots and Egger’s tests were used to evaluate publication bias. We conducted a leave-one-out sensitivity analysis to evaluate the influence of each study on our overall results.

## 3. Results

### 3.1. Characteristics of the Included Studies and Patients

**Figure 1:**
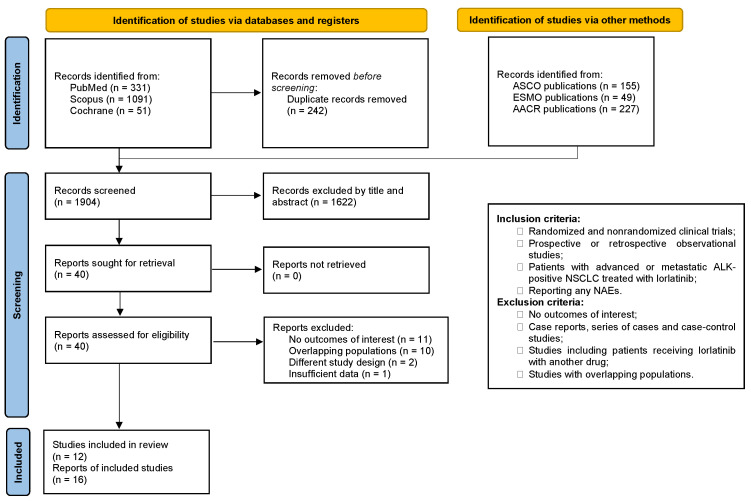
PRISMA flow diagram of study screening and selection.

Figure 1 shows the results of our initial literature search, which returned 1904 results. Forty studies have been identified and fully reviewed in accordance with the inclusion criteria after removing duplicate records and ineligible studies. We included 12 studies and 16 publications involving 1147 patients [8,22,23,24,25,26,27,28,29,30,31,32,33,34,35,36]. Table S4 in the Supplementary Materials contains a complete list of the studies excluded after a thorough analysis.

The study population consisted of individuals diagnosed with advanced or metastatic NSCLC who harbor ALK or ROS1 rearrangements and were treated with lorlatinib, a third-generation TKI. Median follow-up ranged from 6.8 to 23.3 months. Most patients (84%, 959/1147) had a tumoral ALK rearrangement, whereas a smaller proportion (16%, 187/1147) had an ROS1 rearrangement.

Among the studied population, 56% (644/1147) were female, 24% (273/1147) reported a history of smoking, and 33% (381/1147) identified as Asians. In addition, a total of 8.6% (99/1147) of patients had an ECOG performance status of 2 or higher. A history of CNS metastasis was observed in 62% (712/1147) of the patients, whereas 37% (428/1147) did not have a history of CNS metastasis at the initiation of lorlatinib. Also, about 60% (275/476) of patients with brain metastasis received brain-directed radiotherapy. Table 1 displays the main characteristics of the studies included in our meta-analysis, and the publications associated with primary clinical trials.

About 84% (966/1147) of patients received lorlatinib as a second or later line of therapy; 82% (945/1147) were previously exposed to one or more lines of ALK-TKI. Among them, 77% (733/945) were previously treated with a first-generation ALK-TKI, and 46% (532/945) had a prior second-generation ALK-TKI. Supplementary Table S5 shows the past treatment history of patients included in the study.

### 3.2. NAEs in Advanced or Metastatic ALK- or ROS1-Positive NSCLC Patients Receiving Lorlatinib

**Figure 2:**
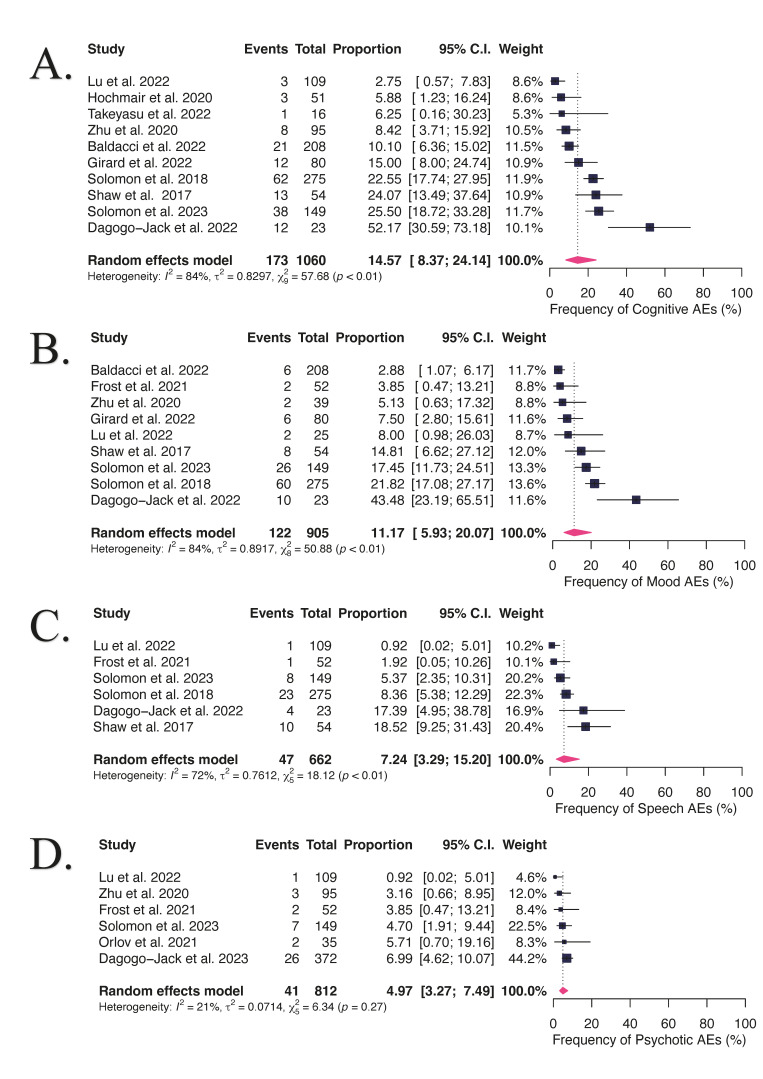
The forest plots depicted a frequency of 14.57% for cognitive AEs (**A**), 11.17% for mood AEs (**B**), 7.24% for speech AEs (**C**), and 4.97% for psychotic AEs (**D**). Ten studies were included for the meta-analyses of cognitive effects [8,22,23,25,26,27,29,31,32,37], nine studies for mood effects [8,22,23,24,25,27,29,32,37], six studies for speech effects [8,23,24,27,29,37], and six studies for psychotic effects [8,23,24,27,28,32].

In a pooled analysis, shown in Figure 2A–D and Supplementary Figure S1A,B, the frequency of cognitive adverse effects (AEs) was 14.57% (95% CI, 8.37 to 24.14, I^2^ = 84%), with a grade 3 or 4 rate of 17.46% (95% CI, 6.75–31.82, I^2^ = 74%). The occurrence of mood AEs was 11.17% (95% CI, 5.93 to 20.07, I^2^ = 84%), with a frequency of grade 3 or 4 of 13.38% (95% CI, 0.68–38.30, I^2^ = 77%). Changes in speech were reported in 7.24% of patients (95% CI, 3.39 to 15.20, I^2^ = 72%). The rate of psychotic AEs was 4.97% (95% CI, 3.27 to 7.49, I^2^ = 21%). Most NAEs were grades 1–2 with no grade 5 reported.

**Figure 3:**
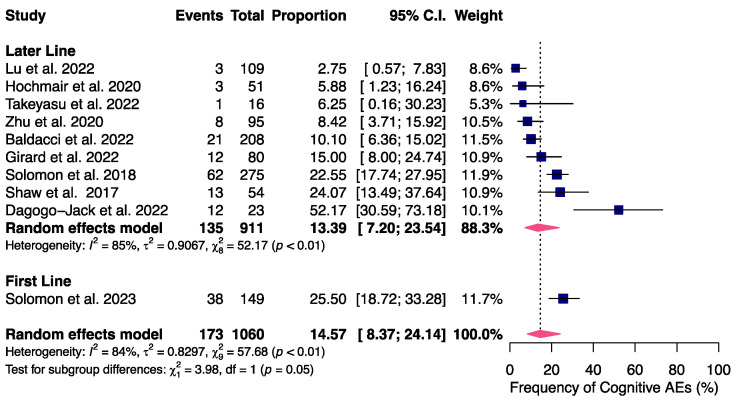
Subgroup analysis of frequency of cognitive AEs according to line of therapy. Patients who received lorlatinib in a first-line [8] setting had a frequency of cognitive AEs of 25.50%, significantly higher than patients receiving lorlatinib in a later line [22,23,25,26,27,29,31,32,37], 13.39% (*p* = 0.05).

Upon further investigation into cognitive and mood AEs, seen in more than 10% of patients, we observed that cognitive effects were more common with lorlatinib administered as first-line compared to later lines of therapy. The rate of cognitive AEs in the first-line setting was 25.5% versus 13.39% in subsequent lines of therapy (*p* = 0.05), as depicted in Figure 3. The frequency of mood AEs did not differ significantly by line of therapy (Supplementary Figure S2).

**Figure 4:**
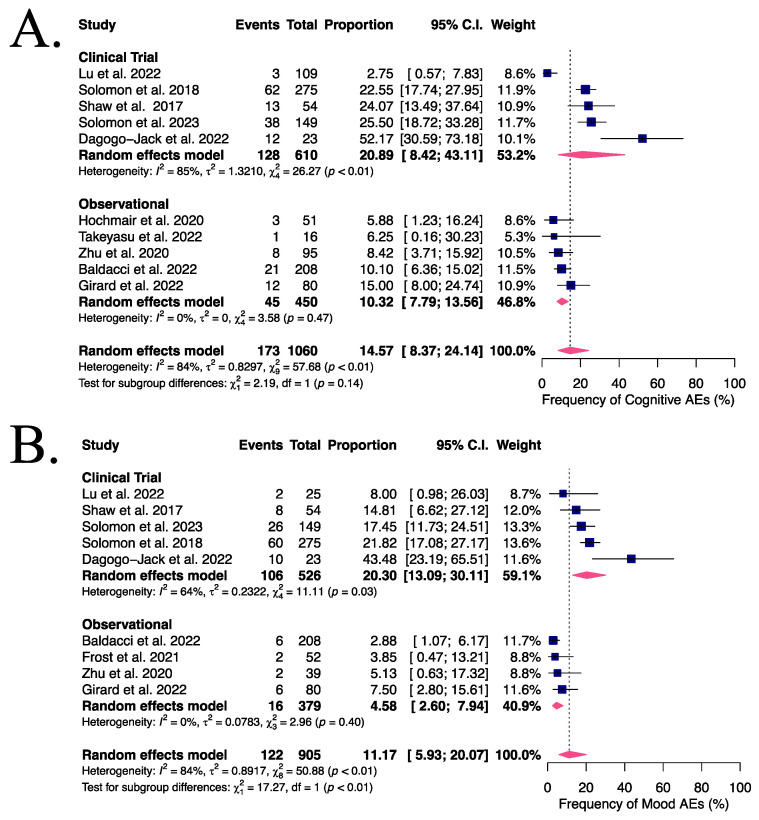
Frequency of cognitive and mood AEs according to study type. Patients who received lorlatinib in a clinical trial [8,23,27,29,37] had frequencies of cognitive (**A**) and mood (**B**) AEs of 20.89% and 20.30%, respectively. In mood effects, this frequency was significantly higher than in the subgroup of observational cohort studies [22,24,25,26,31,32], with mood AEs reported in 4.58% of patients in these studies (*p* < 0.01).

In the subgroup analysis by study type (clinical trials versus real-world evidence), Figure 4A,B, mood AEs revealed a statistically significant subgroup difference (*p* < 0.01). The frequency of mood AEs was higher (20.30%) in clinical trials than in observational cohort studies (only 4.58%). Additionally, heterogeneity was significant in the subgroup of clinical trials (I^2^ = 64%), but not in the observational studies (I^2^ = 0%).

In contrast, this subgroup difference was not observed in cognitive AEs (*p* = 0.4). Interestingly, heterogeneity was also high across clinical trials (I^2^ = 85%), whereas real-world studies were more homogeneous (I^2^ = 0%).

No differences in NAE frequency were identified between patients reported as Asian versus non-Asian, or according to tumor mutation status, ALK-positive versus ROS1-positive NSCLCs, or by the presence versus absence of CNS metastases (Supplementary Figures S3–S5).

### 3.3. Management of NAEs

**Figure 5:**
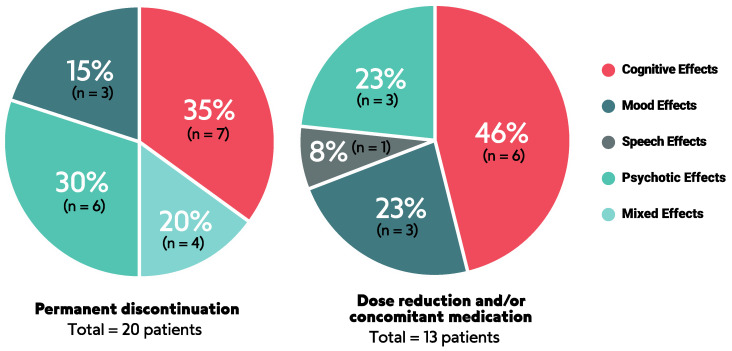
Management of lorlatinib-related NAEs. Mixed effects = mixed NAEs or NAEs with other adverse events.

Management strategies for lorlatinib-related NAEs included permanent discontinuation, dosage modification, and/or adding concomitant medication. We summarized how investigators dealt with lorlatinib-related NAEs in Figure 5, although limited information was available as just eight studies reported this information [8,22,24,25,27,29,30,32].

### 3.4. Quality Assessment

Our findings involve information gathered from five clinical trials and seven observational cohort studies. The randomized clinical trials were considered to have a low risk of bias. Nonrandomized clinical trials were considered to have a moderate and a low risk of bias. Observational cohort studies were assessed using the NOS scale, with scores assigned of 6 or 7 out of 9, except for two observational studies, which scored 4 and 5 out of 9. These lower scores were attributed to a lack of detailed information about the selection process and exposure factors in the study publications [26,28]. The evaluation of randomized and nonrandomized interventional studies and observational cohort studies can be found in Supplementary Figure S6A,B and Supplementary Table S6, respectively.

During the visual examination of the two funnel plots depicting the frequency of cognitive and mood AEs, we observed similar patterns, illustrated in Figure S7A,B. In the cognitive effects and mood effects plots, a predominantly symmetrical pattern was exhibited. Egger’s regression test was conducted to further investigate the matter, which revealed the absence of significant publication bias in both of the two outcomes (cognitive adverse events: *p* = 0.1915 and mood adverse events: *p* = 0.0930).

Leave-one-out sensitivity analyses were conducted to assess the potential influences of each study on the findings. Each study was systematically removed from the pooled estimates in the two variables with the highest frequency: cognitive and mood effects (Supplementary Figure S8A,B, respectively). We did not find any single study that exclusively contributed to the significant heterogeneity observed in our analyses. Overall, the implementation of the leave-one-out test did not significantly modify the outcomes. However, one study [27] appeared to have a slightly positive effect-size influence in cognitive effects, and one study [23] showed a negative influence on mood effects.

## 4. Discussion

In this systematic review and meta-analysis of twelve studies, including 1147 patients, we assessed the frequency of NAEs in advanced or metastatic ALK/ROS1-positive NSCLC patients treated with lorlatinib. The main findings include the following: (1) among NAEs related to lorlatinib, cognitive and mood effects arose as important events with a frequency higher than 10% each; (2) speech effects and psychotic effects had a frequency of about 7% and 5%, respectively; (3) mood AE reports were significantly higher in clinical trials than in real-world studies; and (3) NAEs were well managed with lorlatinib dose reduction and concomitant supportive medications.

To our knowledge, previous high-quality systematic reviews and meta-analyses have not investigated the wide spectrum of lorlatinib-induced NAEs [12,13]. In our analysis, we identified over 1000 patients from several clinical trials and observational cohort studies and investigated the frequency of NAEs. Our population consisted mostly of female, non-smoking, ALK-positive NSCLC patients, and patients with CNS metastasis. These characteristics are reported in previous studies and represent well patients with ALK- and ROS1-positive NSCLC [5,38,39,40,41].

NAEs related to lorlatinib have been associated with the drug’s ability to penetrate the blood–brain barrier and affect the CNS, causing important symptoms that may impair NSCLC patients’ quality of life [9,42]. These adverse events encompass a wide range of signs and symptoms included in the cluster terms of cognitive effects, mood effects, speech effects, and psychotic effects [9]. Additionally, other CNS adverse events, such as headache, insomnia, dysgeusia, and confusion, have also been reported with the use of lorlatinib [9,23].

Lorlatinib can penetrate the brain tissue because of its ability to reduce the number of tight junctions between blood–brain barrier cells [43]. Previous research conducted through in vitro experiments and studies involving mice have provided evidence suggesting that ALK may have a significant impact on the internalization and regulation of the dopamine D2 receptor (D2R). The D2R is expressed in specific regions of the brain that are responsible for controlling motor function, cognition, and motivation. It is hypothesized that inhibiting ALK activity could potentially lead to impairment in D2R desensitization and may be related to neurocognitive impairments [44,45,46].

Interestingly, the frequency of cognitive effects with first-line lorlatinib was higher when compared to patients receiving this drug in a later-line setting. The administration of lorlatinib as a first-line treatment might enhance the spectrum of NAEs, while the first and second generations of TKIs may not adequately induce a threshold to affect the desensitization of D2 receptors. Further investigation is warranted to clarify the molecular modulation of this interaction and identify potential targets to mitigate adverse effects related to the CNS. Our analysis, however, may be biased and underpowered for this conclusion since we identified only one study, open-label, in the first-line setting.

We also demonstrated that the frequency of CNS adverse effects was slightly higher but not significantly different between patients with or without a prior history of brain metastasis. In a cohort of US patients [10], as well as in the CROWN study [7], individuals diagnosed with brain metastasis who underwent brain radiotherapy exhibited an increased frequency of cognitive and speech impairments. These patients have multiple risks for neurocognitive toxicity, including the presence of cerebral metastasis and the toxicity of local therapies that may compromise the blood–brain barrier and increase the risk of NAEs. Thus, it is essential to further investigate the emergence of lorlatinib-related NAEs in individuals with NSCLC, CNS metastatic involvement, and other risk factors for NAEs.

Furthermore, cognitive effects may have an important impact on quality of life. For instance, a clinical trial by Peters et al. performed in 2020 demonstrated a significant deterioration of cognitive function in patients treated with lorlatinib [47]. Similarly, in the CROWN study, crizotinib was found to be more favorable than lorlatinib in terms of cognitive functioning, while lorlatinib was preferred in the physical, emotional, and social domains [48]. Conversely, a recently published observational Dutch study [49] showed that the occurrence of NAEs was not associated with persistent decline in any of the specific neurocognitive domains. This finding was based on extensive neurocognitive assessments and questionaries of 22 patients. Further research is warranted to provide a more comprehensive understanding of lorlatinib’s safety profile in terms of the severity and duration of NAEs.

In a subgroup analysis, we identified that the frequency of mood AEs was greater among patients enrolled in clinical trials compared to those who received lorlatinib through expanded/compassionate access programs in observational cohort studies, reflecting real-world data. We believe these findings are due to the under-reporting of NAEs outside a clinical trial setting. In clinical trials, a rigorous protocol must be followed to report AEs, whereas observational studies might have missing data. The variability in the frequency of reported NAEs across different types of studies may be attributed to the novelty of NAEs in TKIs-treated NSCLC patients and decreased awareness of lorlatinib-related NAEs.

Thus, to understand the complexities of NAEs is fundamental to providing patients with the best benefit in cancer therapy and a good quality of life. Importantly, lorlatinib-related NAEs can be managed through strategies such as dose interruption, dose reduction, or the administration of concurrent supportive medication. In our study, thirty-three patients either permanently discontinued their treatment, underwent dose reduction, and/or received concomitant medications primarily due to cognitive, mood, and psychotic effects. Nevertheless, it is worth mentioning that many studies lack specifics on NAE management, reinforcing the importance of developing support tools for this toxicity profile.

Five clinical trials and seven observational cohort studies were included in this systematic review and meta-analysis. No study was evaluated as having important bias through our prespecified tools for bias assessment in each type of study. In addition, no significant publication bias was found with funnel plot examination and Egger’s testing; nevertheless, Egger’s test may be underpowered for the evaluation of publication bias in terms of mood adverse effects as less than 10 studies were included in this analysis. High heterogeneity was found in the most frequent outcomes of interest (cognitive effects and mood effects), and to further investigate this matter, we performed multiple prespecified subgroup analyses and leave-one-out sensitivity analyses.

The subgroup of observational studies appeared to be homogenous, in contrast to the subgroup of clinical trial data, which may be explained by the inclusion of early-phase trials with small study populations. Moreover, two studies appeared to have a slight effect-size influence on cognitive and mood effects. The positive influence of one study [27] on increasing the frequency of cognitive effects when removed from our analysis may be related to all patients in this study being previously treated with a TKI, which may reflect the attenuated influence of lorlatinib on sensitizing dopamine receptors, as previously discussed. Additionally, the negative influence of a single study [23] on decreasing the frequency of mood effects may be related to all patients in this study having brain metastasis, with some of them being also previously irradiated, which may predispose patients to the development of NAEs [10].

This study has limitations. Initially, we encountered restrictions in our ability to access additional variables related to the occurrence of NAEs, such as pre-existing psychiatric or neurological conditions or the use of psychiatric medications prior to the initiation of lorlatinib. This limitation is due to our reliance on study-level data rather than patient-level data. Also, the detection of cognitive impairment may be more sensitive compared to the detection of other NAEs in the scales employed during the studies.

## 5. Conclusions

In conclusion, our systematic review and meta-analysis presented the overall frequency of lorlatinib-related NAEs and highlighted the high incidence of cognitive and mood events (>10%). The low level of NAEs reported in the real-world data warrants further studies. This may be primarily attributed to differences in patient and healthcare provider awareness, as well as the unfortunate social stigma surrounding mental health conditions and their underdiagnosis. Therefore, our study reinforces the importance of ongoing education for patients, families, and healthcare professionals to enhance their ability to identify any neurocognitive changes in patients undergoing lorlatinib treatment, particularly when patients themselves may not be aware of such alterations.

Fortunately, lorlatinib-related NAEs, when recognized, can be managed with dose interruption, dose reduction, or the administration of concurrent supportive medication. Early diagnosis, management, and follow-up of patients with NSCLC receiving lorlatinib are essential to improve their quality of life.

## Figures and Tables

**Table 1:** Baseline patient characteristics of included studies and associated secondary publications.

Study ID	Study Type	Country	N	Mutation		Sex		Smoking History	Asians	ECOG (>= 2)	CNS Disease n (%)	Brain RT n (%)	Median Follow-Up (mo)
				ALK	ROS1	M	F						
Baldacci et al., 2022 [22]	Ob	France	208	208	-	91	117	64	-	48	160 (77)	95 (46)	23.3
Dagogo-Jack et al., 2022 [23]	CT	USA	23	23	-	13	10	5	5	3	23 (100)	15 (65)	16.8
Frost et al., 2021 [24]	Ob	Germany	52	37	15	24	28	18	-	13	36 (69)	-	16.1
Girard et al., 2022 [25]	Ob	France	80	-	80	33	47	30	-	13	51 (64)	27 (34)	22.2
Hochmair et al., 2020 [26]	Ob	Austria	51	37	14	20	31	20	-	-	28 (55)	-	24.8
Lu et al., 2022 [27]	CT	China	109	109	-	53	56	40	109	5	57 (52)	-	8.4–11.4 *
Orlov et al., 2021 [28]	Ob	Russia	35	35	-	16	19	-	-	-	27 (77)	-	17.5
Shaw et al., 2017 [29]	CT	Multicentric	54	41	12	22	32	-	7	2	39 (72)	27 (50)	17.4
Solomon et al., 2018 [30]	CT	Multicentric	275	228	47	118	157	-	103	10	166 (60)	103 (37)	7.2
Solomon et al., 2023 [8]	CT	Multicentric	149	149	-	65	84	68	65	3	37 (25)	8 (5)	18.3
Takeyasu et al., 2022 [31]	Ob	Japan	16	16	-	8	8	7	16	2	11 (69)	-	12.8
Zhu et al., 2020 [32]	Ob	Multicentric	95	76	19	40	55	21	76	-	77 (81)	-	6.8
Total:			1147	959	187	503	644	273	381	99	712 (62)	275 (60)	-
	Associated publications of main studies												
**Author, Year**		**Related Study ID**						**Analysis Performed**					
Hayachi et al., 2023 [33]		Solomon et al., 2023 [8]						Subgroup analysis for ethnicity					
Soo et al., 2022 [34]		Solomon et al., 2018 [30]						Subgroup analysis for ethnicity					
Shaw et al., 2019 [35]		Shaw et al., 2017 [29], Solomon et al., 2018 [30]						Subgroup analysis for mutation profile					
Bauer et al., 2020 [36]		Solomon et al., 2018 [30]						Subgroup analysis for CNS disease status					

ID: identification. M: male. F: female. ECOG: Eastern Cooperative Oncology Group Performance Status Scale. CNS: central nervous system. CT: clinical trial. Ob: observational. Mo: months. * Study with two separate cohorts.

## Data Availability

The authors confirm that the data supporting the findings of this study are available within the article and its Supplementary Materials.

## Supplementary Materials

The following supporting information can be downloaded at https://www.mdpi.com/article/10.3390/cancers16142611/s1, Table S1: PRISMA 2020 Checklist; Table S2: PRISMA 2020 for Abstract Checklist; Table S3: Definitions of signs and symptoms of the cluster terms cognitive, mood, speech, and psychotic effects based on the Preferred Terms under the Medical Dictionary for Regulatory Activities (MedDRA) Queries; Table S4: Full list of excluded studies after a comprehensive analysis; Table S5: Information on lorlatinib patients’ previous therapies; Figure S1: Grade 3 and grade 4 breakdown analysis of frequency of cognitive (A) and mood (B) AEs; Figure S2: Subgroup analysis of frequency of mood AEs according to the administration setting; Figure S3: Subgroup analysis of frequency of cognitive (A) and mood (B) AEs according to ethnicity; Figure S4: Subgroup analysis of frequency of cognitive (A) and mood (B) AEs according to different genetic mutations; Figure S5: Subgroup analysis of frequency of any CNS AE according to baseline history of CNS disease; Table S6: Characteristics of the interventions on lorlatinib-related NAEs; Figure S6: Bias assessment through Rob2 for randomized interventional studies (A) and ROBINS-I for nonrandomized interventional ones (B); Table S7: Individual study appraisal for assessing the quality of nonrandomized studies in meta-analyses using the Newcastle–Ottawa Scale (NOS); Figure S7: Funnel plots for publication bias analyses of cognitive AEs (A) and mood AEs (B) rates; Figure S8: Leave-one-out test analyses of cognitive AEs (A) and mood AEs (B) rates.
