# Supplementary material for: Neurocognitive Adverse Events Related to Lorlatinib in Non-Small Cell Lung Cancer: A Systematic Review and Meta-Analysis
Source: Cancers (Basel). 2024 Jul 22;16(14):2611. doi: 10.3390/cancers16142611 (PMC11275246; doi:10.3390/cancers16142611)
Supplement: Supplementary file 1 [file cancers-16-02611-s001.zip › cancers-3044071-supplementary.pdf]

SUPPLEMENTARY MATERIALS

Table S1. PRISMA 2020 Checklist. *2*

Table S2. PRISMA 2020 for Abstract Checklist. *6*

Table S3. Definitions of signs and symptoms of the cluster terms cognitive, mood, speech, and psychotic effects based on the Preferred Terms under the Medical Dictionary for Regulatory Activities Query (MedDRA). *7*

Table S4. Full list of excluded studies after a comprehensive analysis. *8*

Table S5. Information on lorlatinib patient's previous therapies *11*

Figure S1. Grade 3 and grade 4 breakdown analysis of frequency of cognitive (A) and mood (B) AEs. *12*

Figure S2. Subgroup analysis of frequency of mood AEs according to the administration setting. *13*

Figure S3. Subgroup analysis of frequency of cognitive (A) and mood (B) AEs according to ethnicity. *14*

Figure S4. Subgroup analysis of frequency of cognitive (A) and mood (B) AEs according to different genetic mutations. *15*

Figure S5. Subgroup analysis of frequency of any CNS AE according to baseline history of CNS disease. *16*

Figure S6. Bias assessment through Rob2 for randomized interventional studies (A) and ROBINS-I for non-randomized interventional ones (B). *17*

Table S6. Individual study appraisal for assessing the quality of nonrandomized studies in meta-analyses using The Newcastle-Ottawa Scale (NOS). *18*

Figure S7. Funnel plots for publication bias analyses of cognitive AEs (A) and mood AEs (B) rate. *19*

Figure S8. Leave-one-out test analyses of cognitive AEs (A) and mood AEs (B) rate. *20*

Table S1. PRISMA 2020 Checklist.

Section and Topic	Item #	Checklist item	Location where item is reported
TITLE			
Title	1	Identify the report as a systematic review.	Page 1
ABSTRACT			
Abstract	2	See the PRISMA 2020 for Abstracts checklist.	Supplementary Materials: Table S2.
INTRODUCTION			
Rationale	3	Describe the rationale for the review in the context of existing knowledge.	Pages 4-5
Objectives	4	Provide an explicit statement of the objective(s) or question(s) the review addresses.	Pages 4-5
METHODS			
Eligibility criteria	5	Specify the inclusion and exclusion criteria for the review and how studies were grouped for the syntheses.	Pages 5-6
Information sources	6	Specify all databases, registers, websites, organizations, reference lists and other sources searched or consulted to identify studies. Specify the date when each source was last searched or consulted.	Page 5
Search strategy	7	Present the full search strategies for all databases, registers, and websites, including any filters and limits used.	Page 5
Selection process	8	Specify the methods used to decide whether a study met the inclusion criteria of the review, including how many reviewers screened each record and each report retrieved, whether they worked independently, and if applicable, details of automation tools used in the process.	Pages 5-6
Data collection process	9	Specify the methods used to collect data from reports, including how many reviewers collected data from each report, whether they worked independently, any processes for obtaining or confirming data from study investigators, and if applicable, details of automation tools used in the process.	Pages 5-7
Data items	10a	List and define all outcomes for which data were sought. Specify whether all results that were	Pages 5-7

Section and Topic	Item #	Checklist item	Location where item is reported
		compatible with each outcome domain in each study were sought (e.g. for all measures, time points, analyses), and if not, the methods used to decide which results to collect.	
	10b	List and define all other variables for which data were sought (e.g. participant and intervention characteristics, funding sources). Describe any assumptions made about any missing or unclear information.	Page 6
Study risk of bias assessment	11	Specify the methods used to assess risk of bias in the included studies, including details of the tool(s) used, how many reviewers assessed each study and whether they worked independently, and if applicable, details of automation tools used in the process.	Page 6
Effect measures	12	Specify for each outcome the effect measure(s) (e.g. risk ratio, mean difference) used in the synthesis or presentation of results.	Page 6-7
Synthesis methods	13a	Describe the processes used to decide which studies were eligible for each synthesis (e.g. tabulating the study intervention characteristics and comparing against the planned groups for each synthesis (item #5)).	Pages 5-7
	13b	Describe any methods required to prepare the data for presentation or synthesis, such as handling of missing summary statistics, or data conversions.	Pages 5-7
	13c	Describe any methods used to tabulate or visually display results of individual studies and syntheses.	Pages 5-7
	13d	Describe any methods used to synthesize results and provide a rationale for the choice(s). If meta-analysis was performed, describe the model(s), method(s) to identify the presence and extent of statistical heterogeneity, and software package(s) used.	Pages 5-7
	13e	Describe any methods used to explore possible causes of heterogeneity among study results (e.g. subgroup analysis, meta-regression).	Pages 5-7
	13f	Describe any sensitivity analyses conducted to assess robustness of the synthesized results.	Pages 5-7

Section and Topic	Item #	Checklist item	Location where item is reported
Reporting bias assessment	14	Describe any methods used to assess risk of bias due to missing results in a synthesis (arising from reporting biases).	Pages 5-7
Certainty assessment	15	Describe any methods used to assess certainty (or confidence) in the body of evidence for an outcome.	Pages 5-7
RESULTS			
Study selection	16a	Describe the results of the search and selection process, from the number of records identified in the search to the number of studies included in the review, ideally using a flow diagram.	Page 7
	16b	Cite studies that might appear to meet the inclusion criteria, but which were excluded, and explain why they were excluded.	Supplementary Materials: Table S3.
Study characteristics	17	Cite each included study and present its characteristics.	Pages 7-8
Risk of bias in studies	18	Present assessments of risk of bias for each included study.	Page 11
Results of individual studies	19	For all outcomes, present, for each study: (a) summary statistics for each group (where appropriate) and (b) an effect estimate and its precision (e.g. confidence/credible interval), ideally using structured tables or plots.	Pages 9-10
Results of syntheses	20a	For each synthesis, briefly summarize the characteristics and risk of bias among contributing studies.	Page 11
	20b	Present results of all statistical syntheses conducted. If meta-analysis was done, present for each the summary estimate and its precision (e.g. confidence/credible interval) and measures of statistical heterogeneity. If comparing groups, describe the direction of the effect.	Pages 7-11
	20c	Present results of all investigations of possible causes of heterogeneity among study results.	Page 11
	20d	Present results of all sensitivity analyses conducted to assess the robustness of the synthesized results.	Page 11
Reporting biases	21	Present assessments of risk of bias due to missing results (arising from reporting biases) for each synthesis assessed.	Page 11

Section and Topic	Item #	Checklist item	Location where item is reported
Certainty of evidence	22	Present assessments of certainty (or confidence) in the body of evidence for each outcome assessed.	Page 11
DISCUSSION			
Discussion	23a	Provide a general interpretation of the results in the context of other evidence.	Pages 12=14
	23b	Discuss any limitations of the evidence included in the review.	Page 14
	23c	Discuss any limitations of the review processes used.	Page 14
	23d	Discuss implications of the results for practice, policy, and future research.	Page 14
OTHER INFORMATION			
Registration and protocol	24a	Provide registration information for the review, including register name and registration number, or state that the review was not registered.	Page 7
	24b	Indicate where the review protocol can be accessed, or state that a protocol was not prepared.	Page 7
	24c	Describe and explain any amendments to information provided at registration or in the protocol.	Page 7
Support	25	Describe sources of financial or non-financial support for the review, and the role of the funders or sponsors in the review.	Page 16
Competing interests	26	Declare any competing interests of review authors.	Page 15
Availability of data, code and other materials	27	Report which of the following are publicly available and where they can be found: template data collection forms; data extracted from included studies; data used for all analyses; analytic code; any other materials used in the review.	Page 8, 33-37

Table S2. PRISMA 2020 for Abstract Checklist.

Section and Topic	Item #	Checklist item	Reported (Yes/No)
TITLE			
Title	1	Identify the report as a systematic review.	Yes
BACKGROUND			
Objectives	2	Provide an explicit statement of the main objective(s) or question(s) the review addresses.	Yes
METHODS			
Eligibility criteria	3	Specify the inclusion and exclusion criteria for the review.	No
Information sources	4	Specify the information sources (e.g. databases, registers) used to identify studies and the date when each was last searched.	Yes
Risk of bias	5	Specify the methods used to assess risk of bias in the included studies.	No
Synthesis of results	6	Specify the methods used to present and synthesize results.	Yes
RESULTS			
Included studies	7	Give the total number of included studies and participants and summarize relevant characteristics of studies.	Yes
Synthesis of results	8	Present results for main outcomes, preferably indicating the number of included studies and participants for each. If meta-analysis was done, report the summary estimate and confidence/credible interval. If comparing groups, indicate the direction of the effect (i.e. which group is favored).	Yes
DISCUSSION			
Limitations of evidence	9	Provide a brief summary of the limitations of the evidence included in the review (e.g. study risk of bias, inconsistency and imprecision).	No
Interpretation	10	Provide a general interpretation of the results and important implications.	Yes
OTHER			
Funding	11	Specify the primary source of funding for the review.	No
Registration	12	Provide the register name and registration number.	No

Table S3. Definitions of signs and symptoms of the cluster terms cognitive, mood, speech, and psychotic effects based on the Preferred Terms under the Medical Dictionary for Regulatory Activities Query (MedDRA).

**Cluster term**	**Signs and symptons**
Cognitive effects	Memory impairment, disturbance in attention, confusion, amnesia, cognitive disorder, delirium, disorientation, and mental impairment.
Mood effects	Anxiety, depression, affect lability, affective disorder, agitation, irritability, mood altered, anger, bipolar disorder, depressed mood, depressive symptom, euphoric mood, mood swings and stress.
Speech effects	Dysarthria, speech disorder and slow speech.
Psychotic effects	Hallucination and delusion.

Table S4. Full list of excluded studies after a comprehensive analysis.

#	Author, year	Title	Reason for exclusion
1.	Barbieri et al., 2022	Safety profile of tyrosine kinase inhibitors used in non-small-cell lung cancer: An analysis from the Italian pharmacovigilance database	Different study design
2.	Bearz et al. 2022	979P Long-term intracranial safety and efficacy analyses from the phase III CROWN study	Overlapping populations with Solomon et al. 2023
3.	Dagogo-Jack et al. 2023	Factors Associated With Developing Neurocognitive Adverse Events in Patients Receiving lorlatinib After Progression on Other Targeted Therapies	Overlapping population with Dagogo-Jack et al. 2022, Zhu et al. 2020, Shaw et al. 2017 and Solomon et al. 2018
4.	Felip et al. 2021	Intracranial and extracranial efficacy of lorlatinib in patients with ALK-positive non-small-cell lung cancer previously treated with second-generation ALK TKIs	Overlapping populations with Solomon et al. 2018
5.	Laktionov et al., 2021	Efficacy of lorlatinib in the treatment of ALK-positive non-small cell lung cancer patients with progression on crizotinib: personal experience	No outcomes of interest
6.	Lee et al. 2021	Real-world efficacy and safety of lorlatinib in treating advanced ALK-positive non–small cell lung cancer patients	No outcomes of interest
7.	Mazieres et al. 2021	MA11.08 Patient-Reported Outcomes from the Randomized Phase 3 CROWN Study of First-Line lorlatinib versus Crizotinib in ALK+ NSCLC	Overlapping populations with Solomon et al. 2023
8.	Mazieres et al. 2022	Patient-reported outcomes from the randomized phase 3 CROWN study of first-line lorlatinib versus crizotinib in advanced ALK-positive non-small cell lung cancer	No outcomes of interest
9.	Okauchi et al. 2020	Real clinical practice in ALK-rearranged NSCLC patients: A retrospective observational study	Insufficient data
10.	Peled et al., 2020	GLASS: Global lorlatinib for ALK(+) and ROS1(+) retrospective Study: real world data of 123 NSCLC patients	Overlapping populations with Frost et al. 2021 and Baldacci et al. 2022
11.	Peters et al. 2020	Impact of lorlatinib on patient-reported outcomes in patients with advanced ALK-positive or ROS1-positive non-small cell lung cancer	No outcomes of interest
12.	Schmid et al., 2022	Real-World Treatment Sequencing, Toxicities, Health Utilities, and Survival Outcomes in Patients	No outcomes of interest

		with Advanced ALK-Rearranged Non-Small-Cell Lung Cancer	
13.	Seto et al. 2020	lorlatinib in previously treated anaplastic lymphoma kinase-rearranged non–small cell lung cancer: Japanese subgroup analysis of a global study	Overlapping populations with Soo et al. 2022 and Solomon et al. 2018
14.	Shaw et al. 2017	A randomized, open-label comparison of lorlatinib versus crizotinib as first-line treatment for advanced anaplastic lymphoma kinase (ALK)-positive non-small cell lung cancer	No outcomes of interest. Overlapping populations with Solomon et al. 2023
15.	Shaw et al. 2020	First-Line lorlatinib or Crizotinib in Advanced ALK-Positive Lung Cancer	Overlapping populations with Solomon et al. 2023
16.	Sisi et al. 2022	Psychiatric Adverse Reactions to Anaplastic Lymphoma Kinase Inhibitors in Non-Small-Cell Lung Cancer: Analysis of Spontaneous Reports Submitted to the FDA Adverse Event Reporting System	Different study design
17.	Solomon et al. 2020	LBA2 lorlatinib vs crizotinib in the first-line treatment of patients (pts) with advanced ALK-positive non-small cell lung cancer (NSCLC): Results of the phase III CROWN study	No outcomes of interest. Overlapping populations with Solomon et al. 2023
18.	Solomon et al. 2021	1199P Dose modification for the management of CNS adverse events in the phase III CROWN study of lorlatinib in non-small cell lung cancer (NSCLC)	Overlapping populations with Solomon et al. 2023
19.	Solomon et al. 2022	Post Hoc Analysis of lorlatinib Intracranial Efficacy and Safety in Patients With ALK-Positive Advanced Non–Small-Cell Lung Cancer From the Phase III CROWN Study	Overlapping populations with Solomon et al. 2023
20.	Solomon et al. 2022	Abstract CT223: Updated efficacy and safety from the phase 3 CROWN study of first-line lorlatinib vs crizotinib in advanced anaplastic lymphoma kinase (ALK)-positive non-small cell lung cancer (NSCLC)	Overlapping populations with Solomon et al. 2023
21.	Talreja et al., 2019	Use of lorlatinib subsequent to crizotinib in anaplastic lymphoma kinase-positive non-small cell lung cancer: Indian experience	No outcomes of interest
22.	Talreja et al., 2020	lorlatinib in anaplastic lymphoma kinase-positive non-small cell lung cancer: Indian experience	No outcomes of interest
23.	Tse et al., 2020	Longitudinal health utilities, symptoms and toxicities in patients with alk-rearranged lung cancer treated with tyrosine kinase inhibitors: A prospective real-world assessment	No outcomes of interest

24.	Zhou et al., 2021	1197P First-line lorlatinib versus crizotinib in ALK-positive non-small cell lung cancer: Asian subgroup analysis of CROWN	No outcomes of interest. Overlapping populations with Solomon et al. 2023

Table S5. Information on lorlatinib patient's previous therapies.

Study ID	N	Later-line	Received >= 1 line of ALK-TKI	Received a 1st generation ALK-TKI (Crizotinib)	Received a 2nd generation ALK-TKI
Baldacci et al. 2022	208	208	208	194	195
Dagogo-Jack et al. 2022	23	23	23	17	23
Frost et al. 2021	52	52	52	50	-
Girard et al. 2022	80	80	80	80	17
Hochmair et al. 2020	51	51	51	39	37
Lu et al. 2022	109	109	109	93	42
Orlov et al. 2021	35	33	31	17	19
Shaw et al. 2017	54	54	48	43	31
Solomon et al. 2018	275	245	232	111	152
Solomon et al. 2023	149	0	0	0	0
Takeyasu et al. 2022	16	16	16	4	16
Zhu et al. 2020	95	95	95	85	-
**Total:**	1147	966	945	733	532

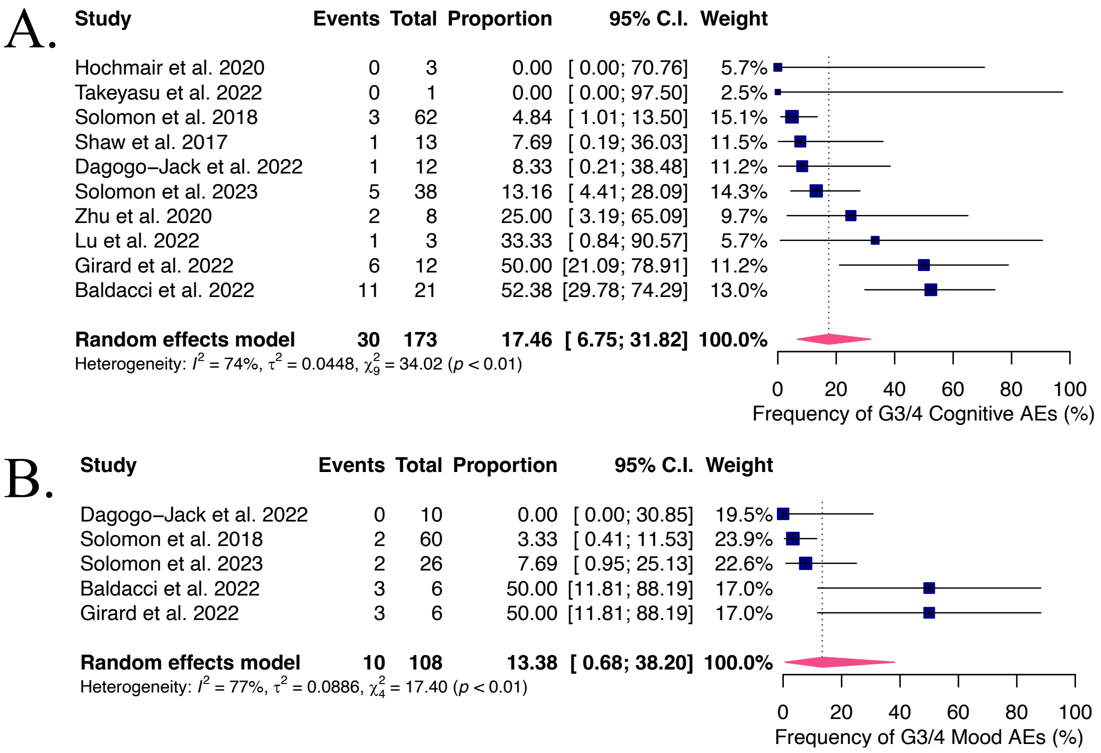

Figure S1. Grade 3 and grade 4 breakdown analysis of frequency of cognitive (A) and mood (B) AEs.

A. The frequency of grade 3 and grade 4 adverse events from all grades cognitive effects was 17.46% (95% CI, 6.75 – 31.82, $I^2 = 74\%$).

B. The frequency of grade 3 and grade 4 adverse events from all grades mood effects was 13.38% (95% CI, 0.68 – 38.20, $I^2 = 77\%$).

Study	Events	Total	Proportion	95% C.I.	Weight
**Later Line**					
Baldacci et al. 2022	6	208	2.88	[1.07; 6.17]	11.7%
Frost et al. 2021	2	52	3.85	[0.47; 13.21]	8.8%
Zhu et al. 2020	2	39	5.13	[0.63; 17.32]	8.8%
Girard et al. 2022	6	80	7.50	[2.80; 15.61]	11.6%
Lu et al. 2022	2	25	8.00	[0.98; 26.03]	8.7%
Shaw et al. 2017	8	54	14.81	[6.62; 27.12]	12.0%
Solomon et al. 2018	60	275	21.82	[17.08; 27.17]	13.6%
Dagogo–Jack et al. 2022	10	23	43.48	[23.19; 65.51]	11.6%
**Random effects model**	**96**	**756**	**10.33**	**[4.99; 20.17]**	**86.7%**
Heterogeneity: $I^2 = 86\%$, $\tau^2 = 1.0251$, $\chi^2_7 = 50.81$ ($p < 0.01$)					
**First Line**					
Solomon et al. 2023	26	149	17.45	[11.73; 24.51]	13.3%
**Random effects model**	**122**	**905**	**11.17**	**[5.93; 20.07]**	**100.0%**
Heterogeneity: $I^2 = 84\%$, $\tau^2 = 0.8917$, $\chi^2_8 = 50.88$ ($p < 0.01$)					
Test for subgroup differences: $\chi^2_1 = 1.78$, df = 1 ($p = 0.18$)					

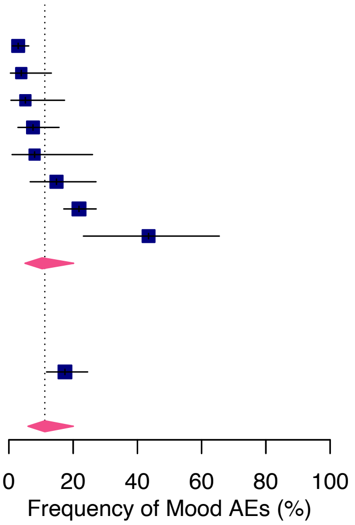

Figure S2. Subgroup analysis of frequency of mood AEs according to the administration setting. Patients who received lorlatinib as first line treatment had a frequency of mood AEs of 17.45% versus 10.33% in patients who received in later line regimens. Statistical significance was not reached ($p = 0.18$).

A.

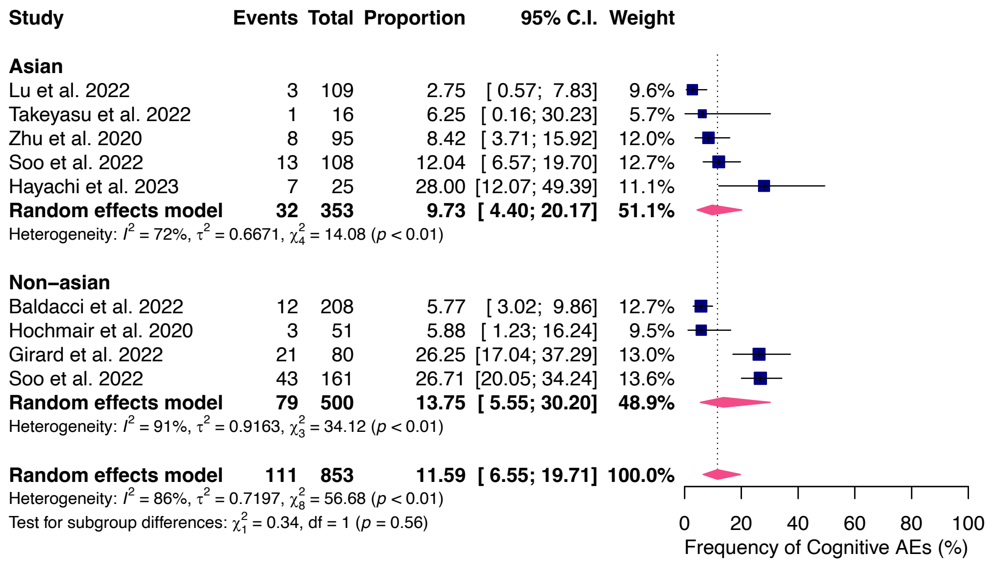

B.

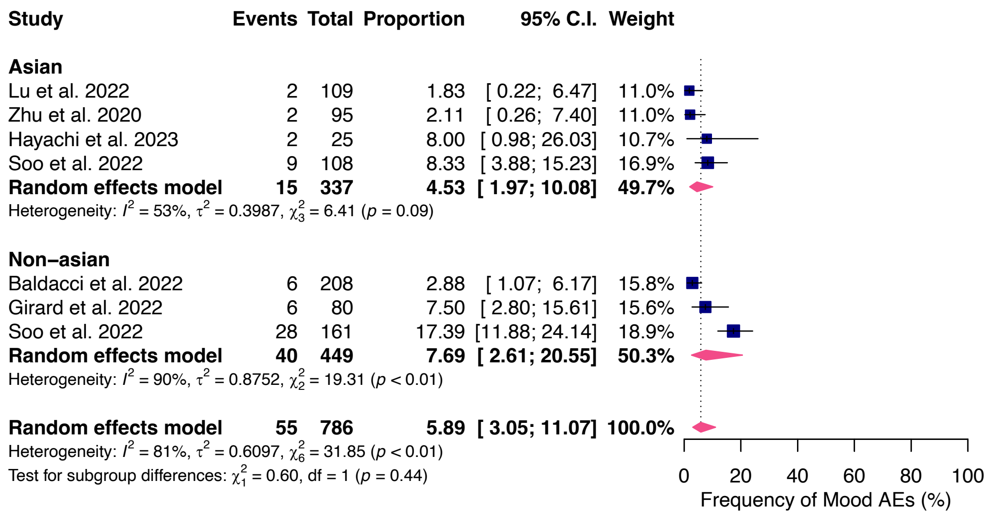

Figure S3. Subgroup analysis of frequency of cognitive (A) and mood (B) AEs according to ethnicity.

A. Non-asian patients had a non-significantly higher frequency of cognitive AEs compared to Asian ones (13.75% versus 9.73%, $p = 0.56$).

B. Non-asian patients had a non-significantly higher frequency of mood AEs compared to Asian ones (7.69% versus 4.53%, $p = 0.44$).

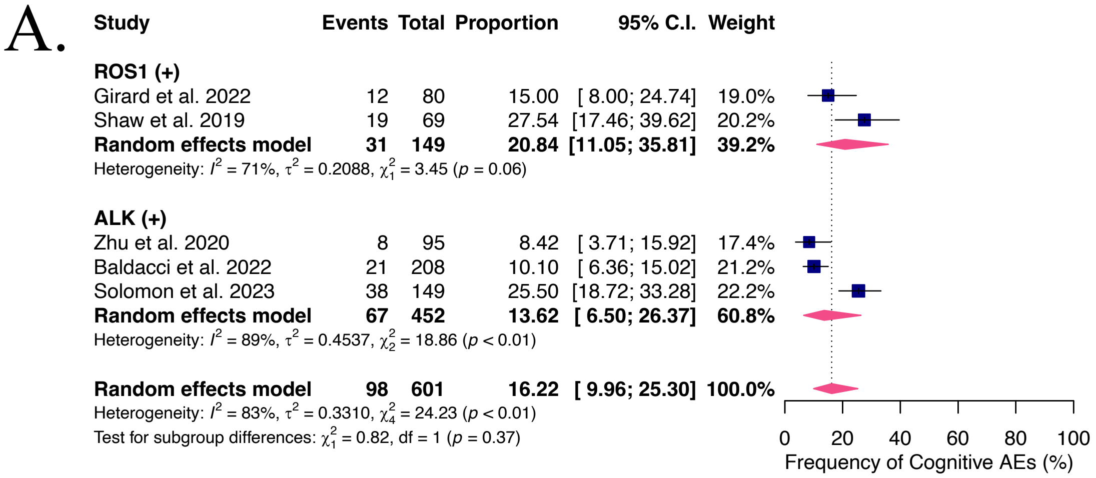

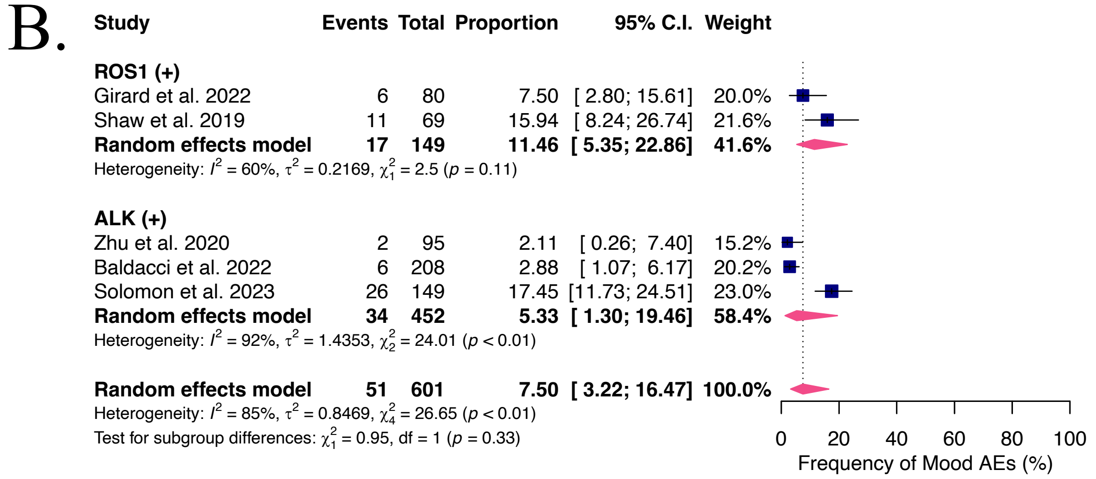

Figure S4. Subgroup analysis of frequency of cognitive (A) and mood (B) AEs according to different genetic mutations.

A. ROS1-positive patients had a non-significantly higher frequency of cognitive AEs compared to ALK-positive ones (20.84% versus 13.62%, $p = 0.37$).

B. ROS1-positive patients had a non-significantly higher frequency of mood AEs compared to ALK-positive ones (11.46% versus 5.33%, $p = 0.33$).

Study	Events	Total	Proportion	95% C.I.	Weight
**With CNS disease**					
Lu et al. 2022	5	57	8.77	[2.91; 19.30]	16.0%
Solomon et al. 2023	16	38	42.11	[26.31; 59.18]	17.1%
Bauer et al. 2020	71	131	54.20	[45.27; 62.93]	18.0%
**Random effects model**	**92**	**226**	**31.36**	**[9.49; 66.56]**	**51.0%**
Heterogeneity: $I^2 = 92\%$, $\tau^2 = 1.5755$, $\chi_2^2 = 25.44$ ($p < 0.01$)					
**Without CNS disease**					
Lu et al. 2022	2	52	3.85	[0.47; 13.21]	13.5%
Solomon et al. 2023	36	111	32.43	[23.85; 41.97]	17.8%
Bauer et al. 2020	33	67	49.25	[36.82; 61.76]	17.6%
**Random effects model**	**71**	**230**	**22.84**	**[4.74; 63.79]**	**49.0%**
Heterogeneity: $I^2 = 89\%$, $\tau^2 = 2.2955$, $\chi_2^2 = 19.03$ ($p < 0.01$)					
**Random effects model**	**163**	**456**	**27.49**	**[12.06; 51.18]**	**100.0%**
Heterogeneity: $I^2 = 90\%$, $\tau^2 = 1.4689$, $\chi_5^2 = 47.95$ ($p < 0.01$)					
Test for subgroup differences: $\chi_1^2 = 0.14$, df = 1 ($p = 0.71$)					

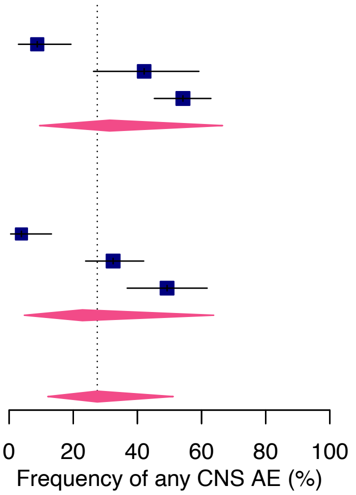

Figure S5. Subgroup analysis of frequency of any CNS AE according to baseline history of CNS disease. Patients who received lorlatinib and had CNS disease had a frequency of CNS AEs of 31.36% versus 22.84% in patients who received it and did not have history of CNS metastasis. Statistical significance was not reached ($p = 0.71$).

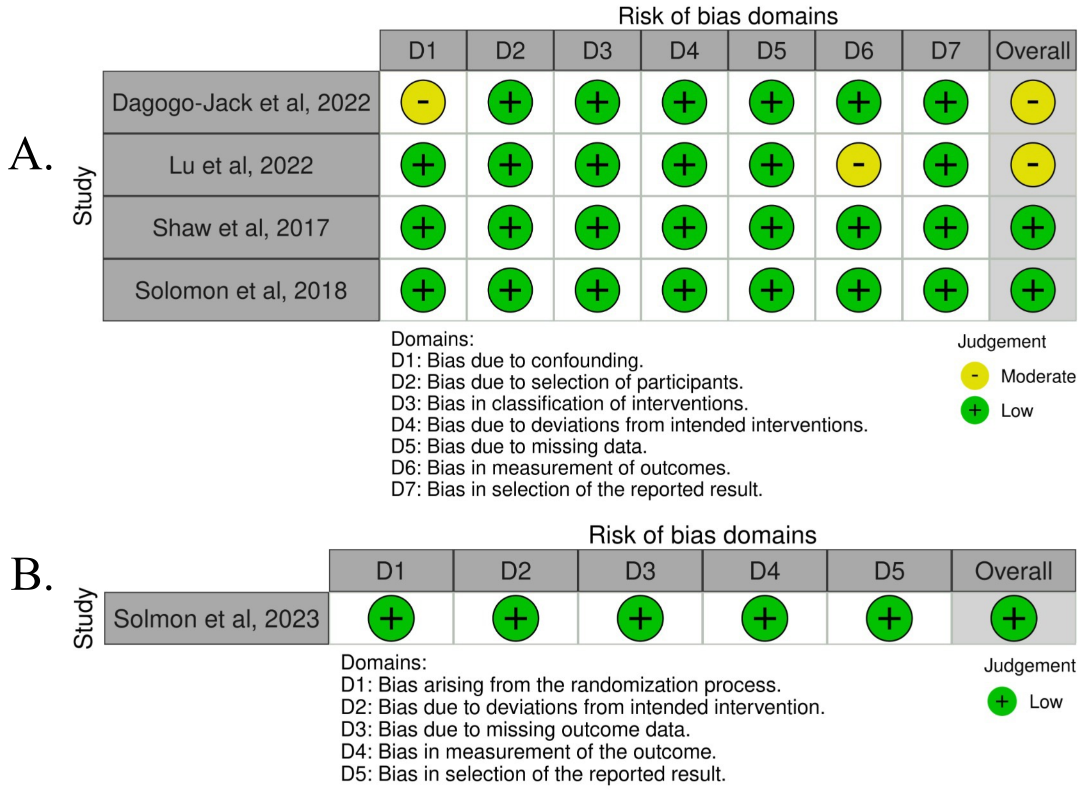

Figure S6. Bias assessment through Rob2 for randomized interventional studies (A) and ROBINS-I for non-randomized interventional ones (B).

Table S6. Individual study appraisal for assessing the quality of nonrandomized studies in meta-analyses using The Newcastle-Ottawa Scale (NOS).

Study ID	Items								
	Selection				Comparability	Exposure			
	1	2	3	4	1	1	2	3	
Baldacci et al, 2022	★	-	★	★	★	-	★	★	**6/9**
Frost et al, 2021	-	-	★	★	★	★	★	★	**6/9**
Girard et al, 2022	★	-	★	★	★	-	★	★	**6/9**
Hochmair et al, 2020	-	-	-	★	★	-	★	★	**4/9**
Orlov et al, 2021	-	-	★	★	★	-	★	★	**5/9**
Takeyasu et al, 2022	-	-	★	★	★	-	★	★	**6/9**
Zhu et al, 2020	★	-	★	★	★	★	★	★	**7/9**

A.

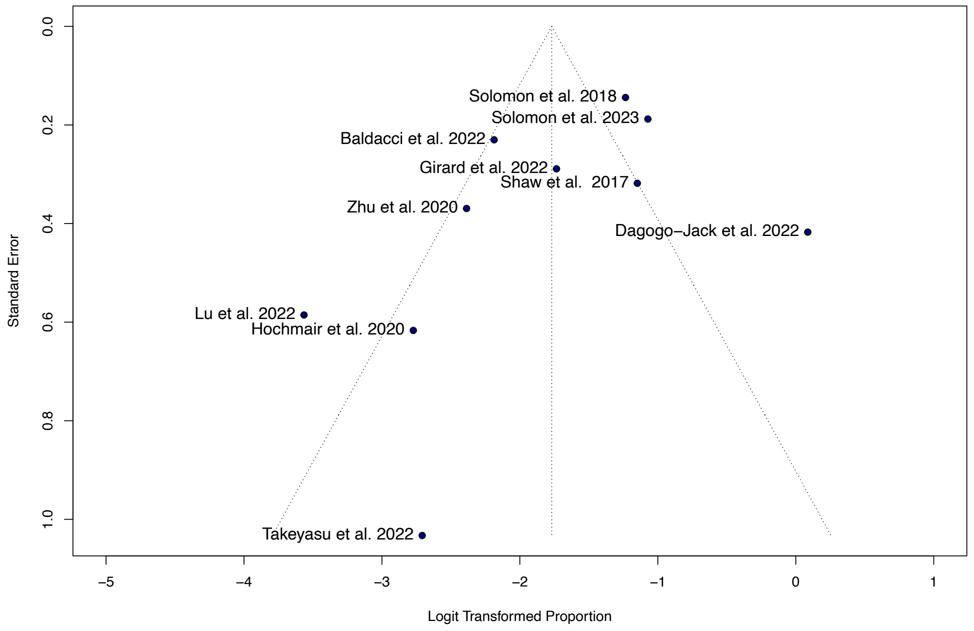

B.

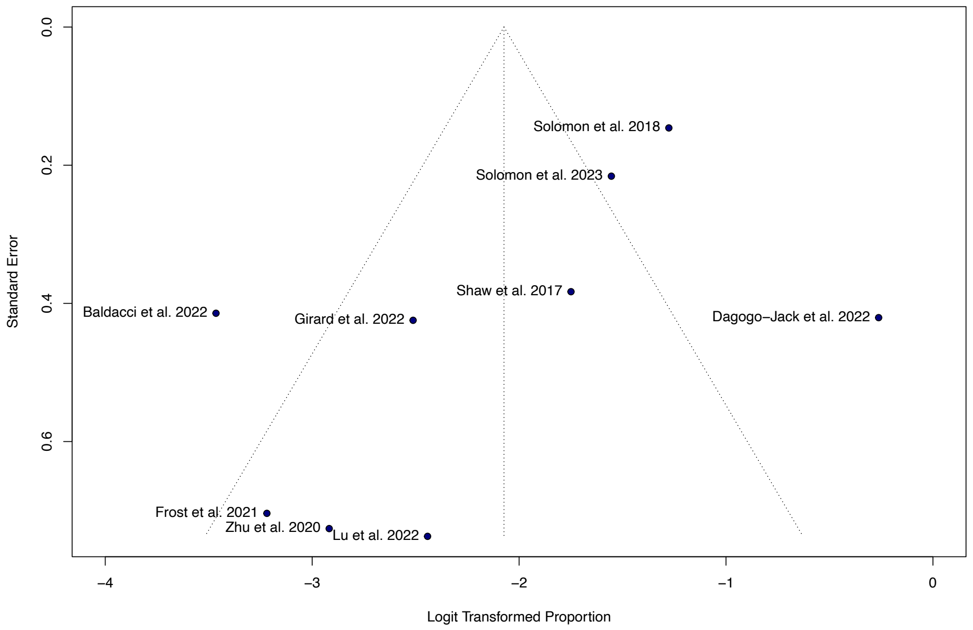

Figure S7. Funnel plots for publication bias analyses of cognitive AEs (A) and mood AEs (B) rate.

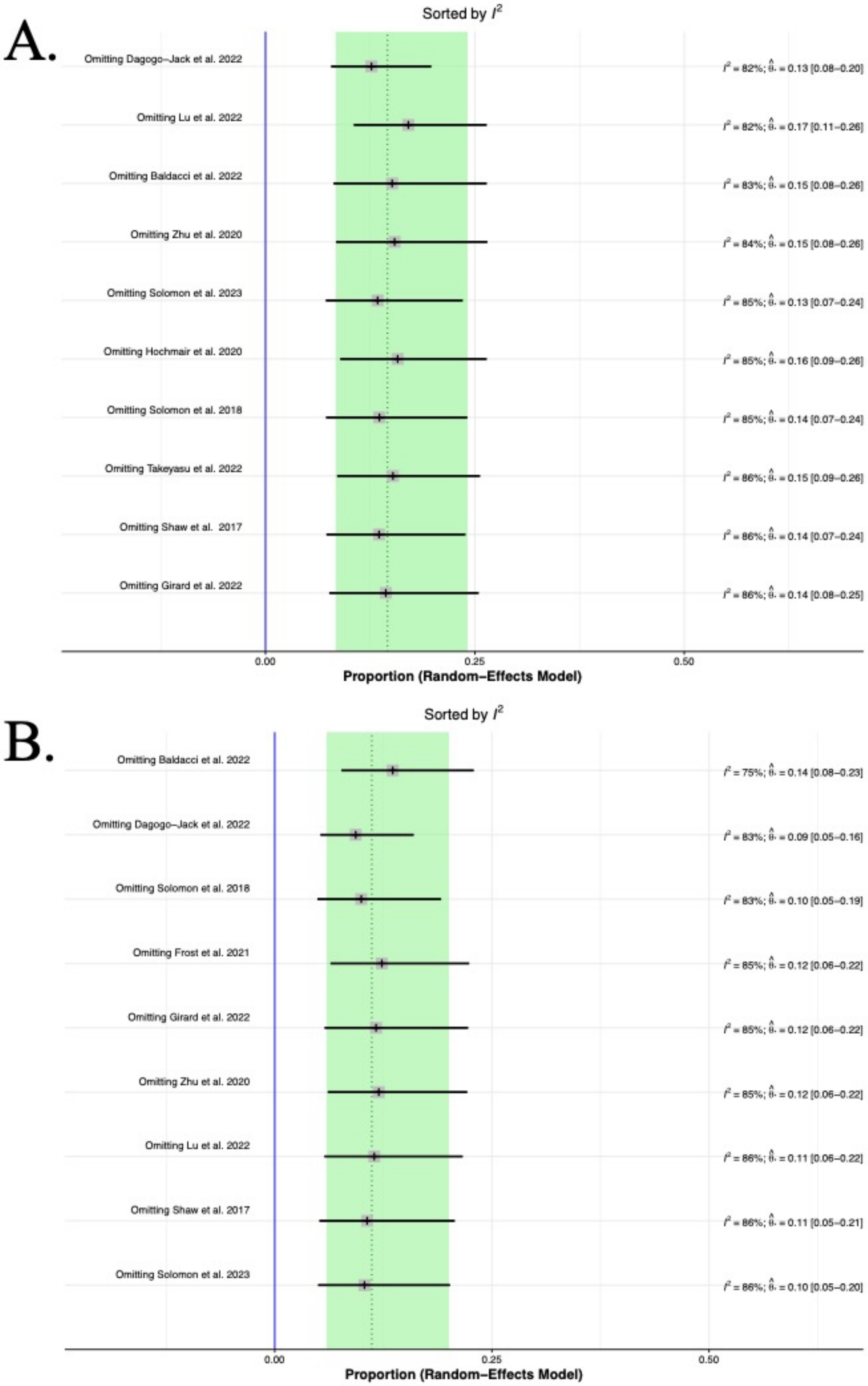

Figure S8. Leave-one-out test analyses of cognitive AEs (A) and mood AEs (B) rate.
